# Trajectories of Rehabilitation across Complex Environments (TRaCE): design and baseline characteristics for a prospective cohort study on spinal cord injury and acquired brain injury

**DOI:** 10.1186/s12913-019-4564-5

**Published:** 2019-10-15

**Authors:** Melissa Legg, Michele Foster, Sanjoti Parekh, Mandy Nielsen, Rachel Jones, Elizabeth Kendall, Jennifer Fleming, Timothy Geraghty

**Affiliations:** 1 The Hopkins Centre: Research for Rehabilitation and Resilience, Menzies Health Institute Queensland, Griffith University and the Division of Rehabilitation, Metro South Health Hospital and Health Service, 199 Ipswich Road, Woolloongabba, Brisbane, Queensland 4102 Australia; 2 School of Health and Rehabilitation Sciences, University of Queensland, Brisbane, Australia

**Keywords:** Acquired brain injury, Spinal cord injury, Health service use, Health service management, Rehabilitation

## Abstract

**Purpose:**

Trajectories of Rehabilitation across Complex Environments (TRaCE), a consented prospective cohort study, addresses a critical need to better understand access to the healthcare system after acute treatment and specialist inpatient rehabilitation for acquired disability. It is expected that this study will produce new knowledge on access to healthcare through the linkage of administrative, survey, and spatial datasets on the one cohort. This paper outlines the study design and baseline characteristics of the cohort.

**Methods:**

The TRaCE cohort is comprised of 165 inpatients who are currently being followed up for 12 months after discharge from specialist rehabilitation for acquired brain injury (ABI) and spinal cord injury (SCI). This project combines a data linkage framework on health service use with a prospective survey on psychosocial wellbeing, geographical information systems to examine spatial accessibility to services, and qualitative interviews with a sub-cohort on experiences of service access.

**Conclusion:**

Ultimately, TRaCE will have strong translational impact on strategies for more targeted interventions to improve the healthcare system and support individuals with acquired disabilities in the long-term.

## Background

Acquired disability is a substantial burden on health systems. Traumatic injury is a leading cause of the global burden of disease and accounts for 300 million disability-adjusted life years [1, 2]. The onset of non-traumatic acquired disabilities also results in a high burden of care owing to the physical, cognitive, or psychosocial impairments incurred [2, 3]. Adults with acquired brain injury (ABI) and spinal cord injury (SCI) are complex populations in terms of their long-term rehabilitative and healthcare needs following acute treatment. Specialised inpatient rehabilitation programs for adults with ABI and SCI are typically provided in dedicated units with a focus on improving functional outcomes. In the transition from hospital to community and beyond, the long-term recovery of functioning and psychosocial wellbeing relies significantly on access to a mix of specialist and mainstream health services in the community such as general practice, pharmacy, physiotherapy, psychology, occupational therapy, speech pathology, or social work [4–6]. This mix of services is not easily determined due to the heterogeneity of the individual recovery process [7–9]. Furthermore, individuals with acquired disability face disadvantages in access to healthcare globally.

Historically, healthcare systems across the world have failed to adequately meet the needs of people with acquired disability who require protracted treatment and ongoing care, with inequitable access being a major issue and priority for reform [10–12]. The World Health Organization [10, 12] identified that people with disabilities face shortfalls in access to healthcare in many countries, including both developing and developed, such as the inequitable geographical distribution of services. Disability is associated with considerable social disadvantage [13, 14] and lower economic resources which also create the conditions for poorer access to healthcare such as residing in geographically disadvantaged areas where services are limited or service systems are less developed [10, 12, 13, 15–17]. In developed countries such as Canada, United States, and Australia this manifests as a shortage of appropriate services in regional or remote areas [10, 12]. People in these areas may face delays to care and unmet needs, which can compromise wellbeing including but not limited to secondary complications, loss of independence and threats to quality of life [5, 18–25]. These disadvantages and disparities epitomize the complex personal and geographical environments that pose considerable barriers to access for people with acquired disability.

Major policy reforms in Australia have been recently introduced, specifically to target systemic failures such as poor access for people with acquired disabilities. The National Injury Insurance Scheme (NIIS) and the National Disability Insurance Scheme (NDIS) focus on providing evidence-based lifetime care and support in a cost-efficient manner for people who acquire long-term disabling conditions. People who sustain ABI and SCI stand to benefit from these Schemes, which also aim for stronger coordination between specialist and mainstream health services. Yet, to realise these benefits, much will depend on identifying and understanding the optimal mix of services [26], taking into account the vastly complex environments which feature variations in the distribution of services and impact access and wellbeing.

While considerable research has been undertaken on health service use amongst people with ABI and SCI, there is limited understanding of the intricate mix of services accessed by cohorts and what access looks like across multiple service systems after discharge from hospital. A common approach for studies conducted worldwide is to describe the use and identify determinants of one type of service such as, for example, hospital emergency department use or inpatient readmissions [27–56]. While this is useful for understanding access to specific types of services, it does not provide information on how services are used relative to others and the patterns of access across time. This type of evidence would be informative for interventions to improve access at the system-level.

Further, it would be of benefit to identify high and low risk access groups at the system-level for intervention. Studies which do describe the mix of services used and patterns across time for individuals with ABI and SCI tend to focus on routinely-collected administrative data [7, 57–62]. Other research has utilised methods which are more appropriate to examine risk in relation to access to healthcare for people with ABI and SCI, such as geographic information system analysis to examine location-based or spatial accessibility to services [17] and survey methods to explore associations with different facets of psychosocial adjustment [6, 63–66]. Arguably, a comprehensive understanding of access to the healthcare system in the aftermath of acquired disability necessitates the aggregation of these data sources and research methods [11, 12]. Exploring relationships between patterns of health service utilisation and psychosocial wellbeing, and geographical location, while relatively new, would be of great value in understanding high and low risk groups and planning services and early intervention.

The current paper outlines the protocol for a multi-component prospective cohort study and reports the baseline characteristics. The project, Trajectories of Rehabilitation across Complex Environments (TRaCE) aims to generate evidence about access to the healthcare system in the 12 months after discharge from specialist inpatient rehabilitation for ABI and SCI. It will identify trajectories of service use with a comprehensive health data linkage framework, complemented by the measurement of spatial accessibility using geographic information system analysis techniques and psychosocial wellbeing using survey methods. Specifically, the objectives of TRaCE are to:

1. Describe the patterns of health service use and unmet needs for the cohort in the 12 months post-discharge.
2. Examine the spatial distribution of available specialist and mainstream health services in relation to the geographical locations of participants and the services they use in the 12 months post-discharge.
3. Identify the injury, personal, social, and spatial determinants of health service use in the 12 months post-discharge.
4. Examine the associations between health service use and psychosocial wellbeing in the 12 months post-discharge.

## Methods

### Recruitment of cohort

The TRaCE cohort is comprised of 165 patients who received specialist inpatient rehabilitation for ABI and SCI at the Division of Rehabilitation, Princess Alexandra Hospital (PAH), located in the Metro South Hospital and Health Service catchment area in the state of Queensland, Australia. The Spinal Injuries Unit (SIU) and Brain Injury Rehabilitation Unit (BIRU) are state-wide specialist services. The 40 bed SIU is the only such unit in Queensland and is the only unit in Australia that provides acute care, specialised inpatient rehabilitation, transitional rehabilitation (i.e. to facilitate the transition from hospital to home), outpatient services and outreach services from one facility. BIRU provides a specialised and dedicated inpatient and Day Hospital services for adults, generally up to 65 years, who sustain an ABI.

**Fig. 1:**
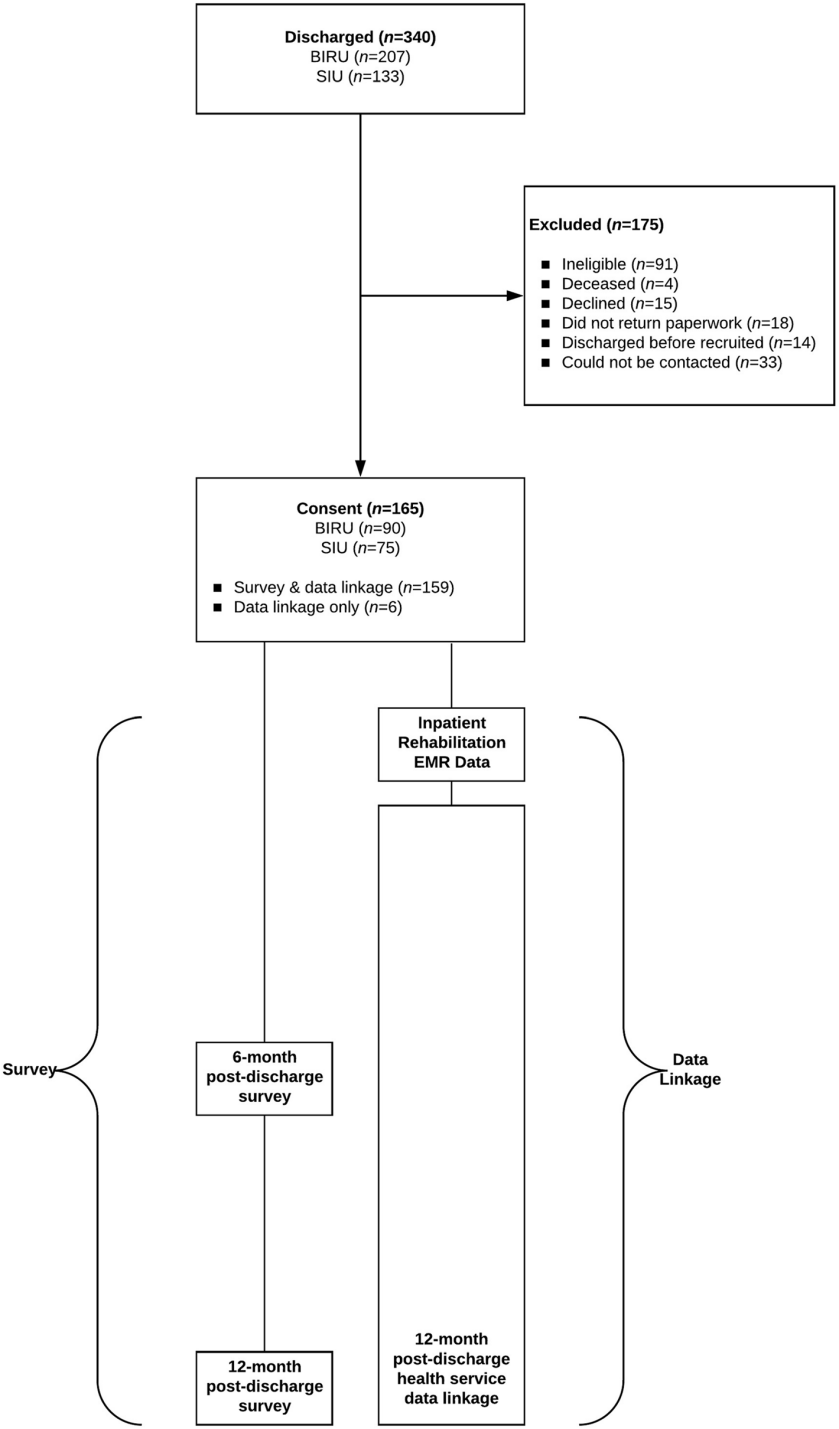
Participant flow of recruitment and follow-up

Ethical approval for this project was granted by the Metro South Human Research Ethics Committee (HREC/16/QPAH/684 SSA/16/QPAH/685) and Griffith University Human Research Ethics Committee (2016/915). Recruitment occurred between March 2017 and March 2018. Eligibility criteria includes: a) a new diagnosis of SCI or ABI documented by a medical practitioner; b) aged 18 years or older; c) capacity to provide informed consent as determined by a medical practitioner or consent by a substitute decision maker on behalf of the individual; and d) communications skills to participate in a survey telephone interview or availability of a substitute decision maker to assist with completion of the survey interview. Eligible inpatients were invited to participate by a designated member of the multidisciplinary treating team. Interested patients were contacted by a member of the research team embedded in the Division of Rehabilitation at PAH to complete the written consent process. The research team contacted a designated substitute decision maker for consent for inpatients with inadequate capacity as assessed by a medical practitioner. Sample size was determined pragmatically rather than by an a priori power calculation. It was dictated by the number of patients seen by the service within the timeframe of the study. Figure 1 outlines the flow of recruitment and the survey and data collection strategy for the TRaCE project.

### Design and follow-up

TRaCE incorporates two key components (Fig. 1). The first component is a prospective cohort study with three types of data: a) a data linkage framework comprised of administrative health records over a 12-month period; b) 6 and 12-month follow-up surveys; and c) spatial mapping and patterning of services with a Geographic Information System. The second component comprises qualitative interviews with a sub-cohort on experiences of service access. At the time of submission to this journal (November 2018), this study was in the process of collecting the 12-month post-discharge data as recruitment, which occurred at the point of discharge from specialist inpatient rehabilitation, was completed in March 2018. Hence, the 12-month follow-up data collection period was expected to last until March 2019.

**Fig. 2:**
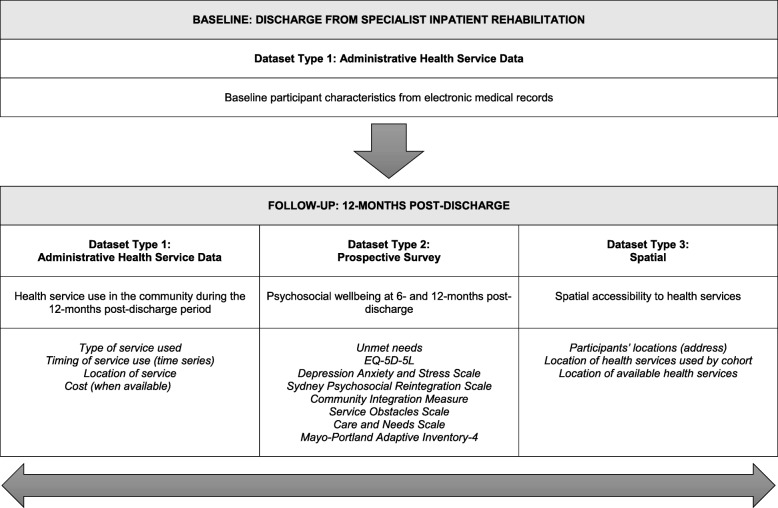
Linkage of datasets

The primary outcome for TRaCE is health service use. Secondary outcomes include psychosocial wellbeing, spatial accessibility to health services, experiences of health service access, and unmet needs. How this data is collected across the different research methods is outlined in Fig. 2.

#### Data linkage framework

The data linkage framework was primarily designed to cover specialist and mainstream health service use in the 12 months after discharge (Fig. 2). Multiple custodians across the Queensland Health Data Linkage Unit were identified for inclusion in this framework. This included emergency services (Emergency Department Information System), hospital inpatient admissions (Queensland Health Admitted Patient Data), and hospital outpatient service use (Queensland Health Non-Admitted Patient Data Collection). General practitioner and prescription medication data were also included in the framework, with the Australian Government Department of Human Services as the designated custodian.

In addition, electronic medical records (EMR) from the PAH were included in the data linkage framework to describe the cohort and identify characteristics of inpatient rehabilitation. Data, which is collected routinely according to Australian Rehabilitation Outcomes Centre (AROC) guidelines [67], includes standard clinical assessment data such as the Functional Independence Measure (FIM) [68].

#### Prospective survey

The prospective survey was incorporated in TRaCE to measure health and psychosocial wellbeing and unmet needs at 6 and 12 months after discharge (Fig. 2). Validated measures of health and psychosocial wellbeing included the EQ-5D [69], Depression Anxiety and Stress Scale [70], Sydney Psychosocial Reintegration Scale [71], Community Integration Measure [72], Service Obstacles Scale [73], Care and Needs Scale [74], and Mayo-Portland Adaptive Inventory [75]. A trained research assistant embedded in the Division of Rehabilitation at PAH was appointed to contact and conduct telephone interviews with consented participants or the nominated proxy when appropriate. Email or postal surveys were included as options at the suggestion of participants.

#### Spatial resources

The spatial component was included in TRaCE to examine spatial accessibility through mapping the location of available services in relation to the geographical location of participants. Geographical information system (GIS) techniques applied in this component were recently piloted by our team [17]. For TRaCE, Esri ArcGIS 10.3 was chosen to geocode and map participants’ addresses. These data are intended to describe the spatial distribution of participants and the services they access during their first 12 months after discharge, with analysis and mapping against the broader distribution of available specialist rehabilitation and mainstream health services. The following geocodable datasets were identified: National Health Services Directory (NHSD), MyHospitals Profile Data, PSMA Australia Limited (PSMA) Localities (May 2015), PSMA Street Network (May 2015), Public transport for Brisbane area, PSMA Airports (Polygon) (August 2014), PSMA Railway Stations (February 2014) and PSMA Railway Lines (August 2014).

#### Qualitative interviews

A qualitative study with semi-structured face-to-face interviews (up to 60 min duration) was developed to collect in-depth information about service use, accessibility, and unmet needs and how these feature under different lifetime care funding schemes (NDIS, NIIS, mainstream, or other). The sub-cohort for this component (approximately 30 participants) was restricted to participants within 150kms of the specialist rehabilitation units. Maximum variation sampling [76] was used to capture varied perspectives, with the primary dimensions of difference including: disability type, gender, and age. Beyond these dimensions, purposive selection was used to recruit participants that varied in terms of type of insurance or disability funding for lifetime support, and geographical location, although this was limited to participants within 150 km of the study site to allow face-to-face interviews to be conducted. The timing of interviews with the sub-cohort was set within the period between 6- and 12-months post-discharge. Options for interview locations included participants’ homes or other preferred community settings. At the time of interview, participants were asked to consent to audio-recording of interviews for transcription and analysis.

### Data analysis strategy

The data analysis strategy was devised to meet project objectives but with enough flexibility to accommodate the exploratory nature of this project. Descriptive statistics (measures of central tendency, frequencies) and plots were included in the strategy to summarise health service use across specified time periods within the 12 months post-discharge. In extension of this project objective, and if appropriate, change in service use will also be explored using longitudinal data analysis techniques which fall within the class of mixed effect models [77]. To explore the relationships between service use and other variables (e.g. psychosocial wellbeing), regression modelling was planned according to the nature of outcome variables including logistic regression for dichotomous outcomes, linear regression for continuous outcomes, and negative binomial or poisson regression models for count outcomes. ABI versus SCI specialist inpatient rehabilitation will be managed by splitting the sample or controlling for this in statistical models using the full sample, when appropriate. Statistical software packages include Stata Version 13, R, and SAS.

For the spatial component, Geographic Weight Regression (GWR), proximity and network analysis and overlay analysis was included in the TRaCE data analysis strategy using Esri ArcGIS 10.3. Visual mapping using ArcMap was also included in the strategy to facilitate interpretation of findings.

Thematic analysis based upon the Framework method [78] was included in the strategy to analyse interview data for the qualitative component. The defining feature of the Framework method is the matrix output which provides a structure to summarise the data.

### Baseline characteristics of cohort

Baseline for the TRaCE cohort was the point of discharge from specialist inpatient rehabilitation. Sociodemographic and impairment characteristics for this cohort are reported in Tables 1 and 2. Aetiologies of injury included road trauma (23.78%), falls (13.42%), sporting and leisure activities (10.98%), assault and intentional self-harm (4.88%), other trauma (5.49%), and other non-trauma (41.46%). The three most common types of comorbidities were cardiovascular conditions (27.27%), mental health or psychiatric conditions (24.24%), and drug and alcohol conditions (21.82%). At baseline the TRaCE cohort had spent, on average, 49.54 days in specialist ABI inpatient rehabilitation (SD = 29.25) or 135.49 days in specialist SCI inpatient rehabilitation (SD = 97.81). At this time, 67.88% of participants were eligible for lifetime care funding. This included funding options to support care needs, equipment, and changes to housing situations in Queensland, Australia such as the NDIS, NIIS Queensland, Workcover, My Aged Care, or the Spinal Cord Injury Response.

**Table 1:** Baseline sociodemographic characteristics

Variable	Total (*n* = 165)	ABI (*n* = 90)	SCI (*n* = 75)
Age (years)^a^			
Mean (standard deviation)	46.18 (17.61)	42.20 (16.65)	50.95 (17.64)
Gender			
Female	26.67%	28.89%	24.00%
Male	73.33%	71.11%	76.00%
Indigenous status			
Aboriginal or Torres Strait Islander origin	2.42%	4.44%	0.00%
Neither Aboriginal nor Torres Strait Islander	95.76%	95.56%	96.00%
Not stated	1.82%	0.00%	4.00%
Marital status			
Married/de facto	47.88%	42.22%	54.67%
Divorced/separated/widowed	16.36%	13.33%	20.00%
Never married	33.33%	41.11%	24.00%
Not stated	2.42%	3.33%	1.33%
Education			
Secondary school	35.15%	42.22%	26.67%
Diploma/certificate/trade/other	23.64%	18.89%	29.33%
Tertiary/postgraduate	16.36%	20.00%	12.00%
Not stated	24.85%	18.89%	32.00%
Labour force status (at time of injury)			
Employed	63.64%	68.89%	57.33%
Unemployed	12.73%	12.22%	13.33%
Student	4.24%	7.78%	0.00%
Not in labour force	3.03%	4.44%	1.33%
Retired	16.36%	6.67%	28.00%

^a^At time of admission to specialist inpatient rehabilitation

**Table 2:** Impairment characteristics at baseline (*n* = 164/165)

Variable	
Stroke	9.76%
Brain dysfunction – Non-traumatic	15.24%
Brain dysfunction – Traumatic	24.39%
Other neurological conditions	1.83%
Spinal cord dysfunction – Non-traumatic paraplegia	14.02%
Spinal cord dysfunction – Non-traumatic tetraplegia	4.88%
Other non-traumatic spinal cord dysfunction	1.22%
Spinal cord dysfunction – Traumatic paraplegia	7.93%
Spinal cord dysfunction – Traumatic tetraplegia	7.93%
Major Multiple Trauma, Brain + spinal cord injury	2.44%
Major Multiple Trauma, Brain + multiple fracture/amputation	3.66%
Major Multiple Trauma, Spinal cord + multiple fracture/amputation	6.71%

n/N indicates missing data

## Discussion

We have outlined the design and baseline characteristics for TRaCE, a prospective cohort study which tracks inpatients with ABI and SCI up to 12 months after discharge from specialist rehabilitation. Ultimately, TRaCE will produce much-needed knowledge about the accessibility of, and actual access to the healthcare system for people who acquire lifelong disabilities. At the forefront of this project is the comprehensive health data linkage framework, which draws together routinely-collected administrative data from multiple custodians that will cover services used in the 12 months after discharge. As such, these data will provide timely insight into what service access actually looks like for people with ABI and SCI after leaving acute inpatient rehabilitation and importantly, the patterns of access over time. The prospective survey will allow wellbeing outcomes to be examined in relation to these access patterns. Further, by mapping the distribution of health services, the geospatial arms will derive information about the accessibility of services across the state of Queensland, Australia. This will also provide the opportunity to examine how TRaCE participants’ trajectories of service use are related to geographical location and concentration of services. Taken together, these data will facilitate an understanding of high and low risk groups in terms of both psychosocial recovery and location. Our team recently piloted the mapping component which identified a mismatch between the supply and demand for rehabilitation services in the Greater Brisbane area in Queensland [17]. Finally, the qualitative component of TRaCE will provide an in-depth understanding of people’s experiences with service access.

While it is generally accepted that, in many jurisdictions, access to both specialist and mainstream services for people who sustain ABI and SCI is not ideal, there is little information available to indicate exactly what type of services are most needed and where. The healthcare and rehabilitation system is, however, replete with routinely-collected data but limited in the knowledge that comes from that data. By combining data from clinical assessments, administrative systems, spatial information and surveys there is greater potential to inform rehabilitation in ways not previously available. By integrating and analysing discrete datasets about healthcare access, a more sophisticated understanding of our rehabilitation populations can be derived to inform service planning and system-level responses.

## Conclusions

In conclusion, TRaCE will produce evidence that will have strong translational impact on strategies for more targeted interventions to improve the healthcare system and support individuals with acquired disability in the long-term. It will identify service and support use trajectories and experiences of rehabilitation with an extensive health data linkage framework that is supplemented by geographic information system analysis techniques, prospective survey methods, and qualitative in-depth interviews. This enables a complete assessment of the impact of inequitable access to healthcare and rehabilitation for people who acquire disabilities and will identify low and high-risk groups for intervention.

## Data Availability

The datasets generated during and/or analysed for the present manuscript are available from the corresponding author on reasonable request.

## Abbreviations

ABI: Acquired brain injury
AROC: Australian Rehabilitation Outcomes Centre
BIRU: Brain Injury Rehabilitation Unit
EMR: Electronic medical record
FIM: Functional Independence Measure
GIS: Geographical information systems
GWR: Geographic Weight Regression
NDIS: National Disability Insurance Scheme
NHSD: National Health Services Directory
NIIS: National Injury Insurance Scheme
PAH: Princess Alexandra Hospital
PSMA: PSMA Australia Limited
SCI: Spinal cord injury
SIU: Spinal Injuries Unit
TRaCE: Trajectories of Rehabilitation across Complex Environments
